# Recent Advances in Explainable Artificial Intelligence for Magnetic Resonance Imaging

**DOI:** 10.3390/diagnostics13091571

**Published:** 2023-04-27

**Authors:** Jinzhao Qian, Hailong Li, Junqi Wang, Lili He

**Affiliations:** 1 Imaging Research Center, Department of Radiology, Cincinnati Children’s Hospital Medical Center, Cincinnati, OH 45229, USA; 2 Department of Computer Science, University of Cincinnati, Cincinnati, OH 45221, USA; 3 Department of Radiology, College of Medicine, University of Cincinnati, Cincinnati, OH 45221, USA

**Keywords:** deep learning, explainable artificial intelligence, magnetic resonance imaging, functional MRI, diffusion MRI, MR angiography, convolutional neural networks, Grad-CAM

## Abstract

Advances in artificial intelligence (AI), especially deep learning (DL), have facilitated magnetic resonance imaging (MRI) data analysis, enabling AI-assisted medical image diagnoses and prognoses. However, most of the DL models are considered as “black boxes”. There is an unmet need to demystify DL models so domain experts can trust these high-performance DL models. This has resulted in a sub-domain of AI research called explainable artificial intelligence (XAI). In the last decade, many experts have dedicated their efforts to developing novel XAI methods that are competent at visualizing and explaining the logic behind data-driven DL models. However, XAI techniques are still in their infancy for medical MRI image analysis. This study aims to outline the XAI applications that are able to interpret DL models for MRI data analysis. We first introduce several common MRI data modalities. Then, a brief history of DL models is discussed. Next, we highlight XAI frameworks and elaborate on the principles of multiple popular XAI methods. Moreover, studies on XAI applications in MRI image analysis are reviewed across the tissues/organs of the human body. A quantitative analysis is conducted to reveal the insights of MRI researchers on these XAI techniques. Finally, evaluations of XAI methods are discussed. This survey presents recent advances in the XAI domain for explaining the DL models that have been utilized in MRI applications.

## 1. Introduction

Advances in artificial intelligence (AI), especially deep learning (DL), have enabled more complex magnetic resonance imaging (MRI) data analysis, facilitating tremendous progress in automated image-based diagnoses and prognoses [1]. Previously, medical image analyses were typically performed using systems fully designed by human domain experts [2]. Such an image analysis system could be a statistical or machine learning (ML) model that used handcrafted properties (i.e., image features) of an image or regions of interest (ROIs) on the image [3]. These handcrafted image features range from low-level (e.g., edges or corners) to higher-level image properties (e.g., texture). Modern DL models can automatically learn these image features with minimal human interference to optimally perform certain image analysis tasks, which improves efficiency and saves a lot of human resources [4]. The fast development of DL has contributed to its growing application in MRI image analysis.

Due to their non-linear underlying structures, most DL models are considered as “black boxes” by scholars, and even more so by the public [5]. There is an urgent need for more tools to demystify these DL models, which has resulted in a sub-domain of AI research called explainable artificial intelligence (XAI). The emergence of XAI has mainly been driven by three factors: (a) the need to increase the transparency of AI models; (b) the necessity to allow humans to interact with AI models; and (c) the requirement for the faithfulness of their inferences. The above reasons have led to the rapid development of domain-dependent and context-specific techniques when dealing with the interpretation of DL models and the formation of explanations for public understanding [6]. Recently, many experts have dedicated their efforts to developing novel methods that are competent at visualizing and explaining the logic behind data-driven DL models.

**Figure 1:**
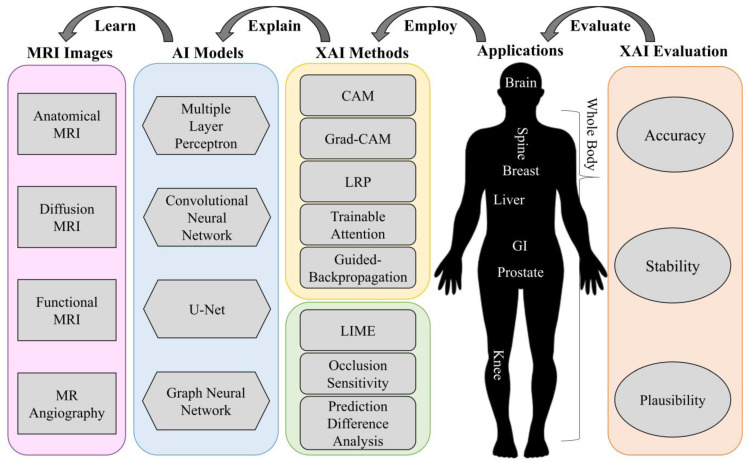
Organization of our survey in this work. We first review MRI images. Next, we introduce common AI models that have been applied to “Learn” those MRI images. Then, we elaborate on popular XAI methods that can “Explain” the classification or segmentation results of the previous AI models. Moreover, we investigate MRI applications that “Employ” AI models and XAI techniques. Finally, we discuss the evaluation metrics that are proposed to “Evaluate” how well these XAI methods explain the AI models.

XAI research has been rapidly growing over the last decade. Multiple high-quality reviews on XAI techniques exist for the computer vision or general AI community [7,8]. In this work, we seek to use a survey to gain insights into recent advances in XAI methods and their applications in MRI analysis. In contrast to those prior XAI reviews, the scope of the current study focuses on the MRI research community, where a systematic view of the whole AI-related pipeline is necessary for rigid MRI research and clinical translation. As such, we strive to introduce the XAI technique from this unique perspective to the MRI research community. We present those pioneering XAI studies from a systematic perspective as a chained pipeline, starting from MRI data to AI models, XAI methods, specific MRI tasks/applications, and all the way to the final XAI evaluation. Figure 1 illustrates the chained pipeline of XAI-related MRI studies. The potential benefits XAI can bring to the field of MRI analysis are huge. XAI techniques not only manage to provide an explanation for AI decisions and pave the way to utilizing informative MRI data, but also improve the transparency and trustworthiness of AI systems in healthcare, which is essential for their widespread adoption and acceptance. The significance of our survey lies in providing the most up-to-date trends of XAI approaches utilized in recent MRI research. The quantitative analysis of those XAI studies reveals the most common XAI techniques used in various MRI studies. We also discuss the strengths and limitations associated with these XAI techniques. Figure 1 also serves as the organization of this survey. In Section 2, we give an overview of MRI images. In Section 3, we briefly introduce popular AI models that have been applied to “Learn” those MRI data. In Section 4, we elaborate on several XAI techniques that can “Explain” the classification or segmentation results of the previous AI models. In Section 5, we further discuss the MRI applications that “Employ” AI models and XAI techniques in MRI research. In Section 6, we discuss the evaluation metrics that are proposed to “Evaluate” the explainability of these XAI methods. This is followed by the conclusion and outlook on XAI in Section 7.

## 2. Overview of MRI Images

**Figure 2:**
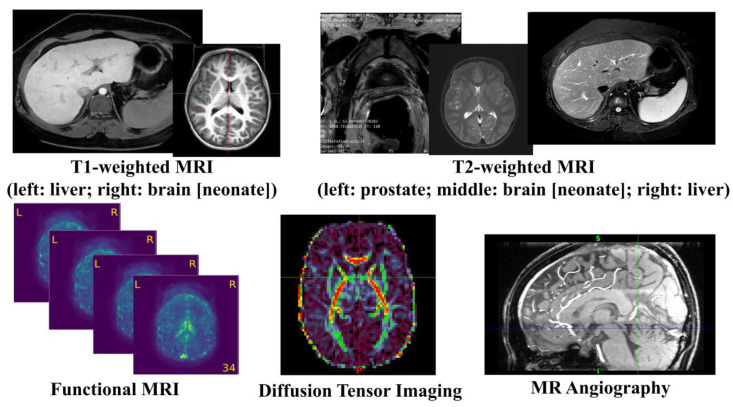
Illustration of common MRI Images.

MRI uses the principle of nuclear magnetic resonance (NMR) [9] and maps the internal structure of an object by acquiring the position and type of its atomic nuclei [10]. The application of gradient magnetic fields leads to the emission of electromagnetic waves based on the attenuation of the energy released in different structural environments within a substance. As a noninvasive imaging technology, MRI can produce high-quality images without the use of ionizing radiation. Thus, MRI can safely provide a wealth of diagnostic information, which makes medical diagnoses and functional studies of the human body convenient and effective [11]. MRI is a versatile medical imaging technique that produces images of organs, tissues, bones, and other structures for a range of medical conditions, and has been widely used in clinical disease screening, diagnosis, treatment guidance, and evaluation since the mid-1980s. Figure 2 illustrates a number of examples using different MRI techniques from various human organs. In this section, we will review a few common MRI techniques that have been widely utilized in both the clinical and research domains.

### 2.1. Anatomical MRI

T1-weighted MRI is one of the most commonly used anatomical MRI sequences using T1 relaxation time [10]. T1 (also known as spin–lattice or longitudinal) relaxation time is the time for the z component of a spin to return to 63% of its original position following a radiofrequency (RF) excitation pulse. Since various tissues require different T1 relaxation times to return to equilibrium, one can highlight the tissues’ contrast using differences in the T1 relaxation times. T2-weighted MRI is another common anatomical MRI sequence, which relies on T2 relaxation time [12]. T2 (also known as spin–spin or transverse) relaxation time is the time required for the transverse component of a proton to decay to 37% of its initial status through irreversible processes [10]. Similar to T1-weighted MRI, various human tissues also have different T2 relaxation times, so we can demonstrate the tissues’ contrast using differences in the T2 relaxation times. T1-weighted images are produced by scans using short Time to Echo (TE) time and Repetition Time (TR). Conversely, T2-weighted images are generated by scans using longer TE and TR time. The contrast and brightness of anatomical MRI images are predominately determined by the T1 and T2 properties of the tissue, separately. While T1-weighted images tend to have a high-signal intensity on fat and low intensity on water, T2-weighted images have an intermediate–high-signal intensity on fat and high intensity on water. For example, T1-weighted MRI images highlight white matter for the adult brain, while T2 MRI images highlight cerebrospinal fluid and inflammation [13].

### 2.2. Diffusion MRI

Diffusion MRI, or diffusion-weighted imaging (DWI), is one MRI technique that generates image contrast by measuring the Brownian motion of the water molecules within tissues. Diffusion Tensor Imaging (DTI), a special type of DWI, is one of the most popular diffusion MRI techniques in brain research and clinical practice for mapping white matter tractography [14]. It measures the diffusion anisotropy of water molecules traveling in white matter fibers, where a higher speed is observed in parallel motion compared to perpendicular movements. By detecting the variations in the signals from hydrogen atoms, DTI can capture the orientations of the white matter tracts in the brain. Quantitative diffusion metrics, such as fractional anisotropy, axial diffusivity, mean diffusivity, and radial diffusivity, have been extensively used in brain research to reveal the white matter integrity. These white matter tracts have found multiple neuroimaging applications, such as brain structural and functional mapping, evaluations of brain injury, disease progression, surgical planning, and treatment response monitoring [15].

### 2.3. Functional MRI (fMRI)

Functional MRI (fMRI) is an imaging technique measuring the time-varying brain activity reflected by the fluctuations of blood oxygen levels caused by brain metabolism [16]. The oxygen is believed to concentrate at the location where the neural activity is highly active. Due to the magnetic sensitivity difference between the oxygenated and deoxygenated hemoglobin, a measurable signal is detected by the MRI scanner. Two types of brain activation patterns can be obtained when subjects are in a resting state (resting state fMRI) or taking on targeted tasks (task fMRI). Since fMRI data are 4D time-varying volume data, graph-based approaches are widely used to construct the brain’s functional connectomes from the fMRI data by estimating the correlations between the distinct brain regions, where each node represents a brain region and the edges represent the functional connections. In recent years, fMRI has been used to investigate a wide range of cognitive tasks, including attention, emotion, working memory, language, and decision making, as well as neurological and psychiatric disorders (e.g., Alzheimer’s disease, attention deficit hyperactivity disorder, and schizophrenia).

### 2.4. Magnetic Resonance Angiography (MRA)

Magnetic resonance angiography (MRA) [17] is a special type of MRI designed to image the vascular system. It plays an essential role in the accurate diagnosis of and treatment selection for patients with arterial disease. Contrast-enhanced (CE) MRA provides more detailed images for more precise diagnoses with shorter acquisition times and reduced artifacts caused by blood flow and pulsatility, but increases examination expenses and the risk of nephrogenic systemic fibrosis caused by gadolinium-based agents. Non-contrast-enhanced (NCE) MRA provides a safer tool for generating image contrasts between blood vessels and background tissues and is becoming increasingly popular in clinical practice. Among the various NCE MRA techniques, time-of-flight (TOF) imaging is the most common and is widely used in clinical practice and research fields [18], which measures the magnetization state difference between stationary tissues and blood flow. TOF MRA has been applied to the assessment, diagnosis, and treatment of multiple cerebrovascular and arterial diseases.

## 3. Brief Introduction of AI Models

Multi-layer perceptron (MLP), also known as artificial neural networks, are one of the most classic ML models [19]. An MLP consists of an input layer, many hidden layers in the middle, and an output layer. Each neuron in an MLP is connected to all the nodes in the previous layer. Since MLPs have a large number of weights in each layer, it is difficult to train these models, especially when the data dimension (such as images) is high. Additionally, as MLPs only accept vectorized features as inputs, they are not a preferrable model for image data that contain spatial information. More recently, deep neural networks (DNN) have been commonly utilized to refer to MLP models with a large number of hidden layers.

Convolutional neural networks (CNN) are the most frequently utilized models for tackling different medical imaging tasks, such as image classification/regression. Different from the fully connected neurons in MLPs or DNNs, CNN models rely on shared local trainable kernels/filters to perform their image convolution operations on the input images to extract the image features. Compared to MLP models, CNN models not only incorporate the spatial location of the shared features within the input data/images, but also have a decreased computational complexity, resulting in less encoding of the overall parameters [20]. Taken together, these characteristics open up the possibility for the application of CNN models to more limited, sparse datasets, as seen in the setting of medical imaging applications. A major milestone in DL history is AlexNet [21], a CNN model that won the ImageNet competition in 2012 with outstanding scores. Since then, multiple CNN models, such as the Visual Geometry Group (VGG) [22], GoogLeNet [23], and Residual Networks (ResNet) [24], have been developed to further improve the capability of image classification and recognition.

For image segmentation, U-Net [25] or its variations become desirable DL models. The principle of U-Net is to use a U-shaped CNN architecture with skip connections to compute attention maps at full input resolution to help in the detection of small objects. More specifically, U-Net, as well as its variation models (e.g., V-Net and ResU-Net), all consist of a contracting path and an expanding path. Each path has the repeated block of convolutional/deconvolutional layers, non-linear activation layers, and pooling layers for feature learning and reconstruction.

Graph neural networks (GNN) [26] generalize DL models on graph-based data. As the most classical and widely used GNN, a graph convolutional network (GCN) [27] has been proposed by Kipf and Welling as an efficient variant of a CNN that performs convolution on graphs. Various variants of GNN models, such as the Graph Isomorphism Network (GIN) [28], Graph Attention Network (GAT) [29], and GraphSAGE [30], have been proposed and adopted to tackle medical image problems at the node level, edge level, and graph level.

## 4. XAI Techniques

In recent years, a number of XAI methods have been proposed to explain the abovementioned DL models. These XAI techniques can be categorized into model-specific explanations and model-agnostic explanations, according to a survey by Adadi and Berrada [7]. Model-specific explanation methods can only be applied to certain specific models. For example, an XAI method may use attributes specific to a type of DL model. On the other hand, model-agnostic explanation methods are independent of DL models, operating solely on the input and output of the DL models. For example, to explain which regions are driving the output, the researchers perturb the input to observe what the change is in the output of the DL models. A distinct advantage of model-specific explanation methods is their computational cost in contrast to model-agnostic explanation methods. This computational cost could be assessed by comparing how these explanation techniques work, even if it is rarely mentioned in papers. Model-specific techniques make a fast single pass back through the neural network, while model-agnostic explanation methods require an extensive perturbation of the input images to measure the change in the output caused by the perturbations. For example, using the Grad-CAM approach, researchers solely require choosing which layer will inspect the activation. On the other hand, model-agnostic techniques rely on relatively complex fine-tuning [31]. Model-agnostic techniques overwhelm model-specific techniques in terms of the potential of XAI techniques to be “plug-and-play” (also known as “ease of use”). Consisting of perturbation-based visual explanation, model agnostic techniques have the highest ease of use, enabling them to be applied to any trained neural network to provide a visual explanation. In the following section, we elaborate on techniques in both the model-specific and model-agnostic categories.

### 4.1. Model-Specific Explanation Methods

#### 4.1.1. Class Activation Mapping (CAM)

Class Activation Mapping (CAM) is one of the early techniques for explaining CNN models by equipping CNNs with remarkable localization ability [32]. It replaces the fully connected layers at the end of a CNN with global average pooling on the last convolutional feature map. The CAM’s heatmap is a weighted linear sum of the presence of visual patterns captured by the filters at different spatial locations, which can be expressed as below:

(1) $$M_{c}\left(x,y\right)=\sum_{k}w_{k}^{c}f_{k}\left(x,y\right),$$

where $f_{k}(x,y)$ refers to the activation of unit k in the final convolutional layer and $w_{k}^{c}$ represents the importance of $f_{k}(x,y)$ for a certain class *c*. A multi-scale CAM method has also been proposed by utilizing the multiple scale information in MRI images. Some studies have concatenated feature maps at the three scales provided as inputs for the global average pooling [33], while other works have concatenated each layer’s feature maps before max pooling, giving these as inputs to the global average pooling layer. The generated activation maps showed a higher resolution than the single-scale maps and provided more accurate localizations of brain tumors in MRI scans [34].

#### 4.1.2. Gradient-Weighted Class Activation Mapping (Grad-CAM)

Gradient-weighted Class Activation Mapping (Grad-CAM), a generalization of the CAM method, is one of the most popular XAI methods for demystifying where CNN models are looking during inference [35]. Grad-CAM uses the gradients of the target concept to flow into the targeted convolutional layer and produces a coarse localization map. By highlighting the important regions in the image, the map makes the prediction of specific labels more transparent. In practice, to visualize Grad-CAM for a category, all the feature maps in the last layer of the CNN are taken as partial derivatives. This is because the last layer is rich in high-level semantic information and detailed spatial information, and partial derivatives represent the rate of change in the output with respect to the input, that is, how much the output changes by one unit on the feature map. The partial derivatives can reflect the output of the degree of sensitivity. If the gradient is large, it will be very sensitive, indicating that the location is more likely to be the target category. In contrast to CAM, Grad-CAM acquires the neuron importance weights via flowing back the gradients that are global-average pooled, calculated as below:

(2) $$\alpha_{k}^{c}=\frac{1}{Z}\sum_{i}\sum_{j}\frac{\partial y^{c}}{\partial A_{ij}^{k}},$$

where $y^{c}$ is the score for class *c* and $A^{k}$ is the feature map of a convolutional layer. The weights that Grad-CAM computes from the global average of the gradients are equivalent to those computed by CAM, whose mathematical derivation can be found in the original paper. Then, the class-discriminative localization map of Grad-CAM can be obtained as below:

(3) $$L_{Grad-CAM}^{c}=ReLU\left(\sum_{k}\alpha_{k}^{c}A^{k}\right).$$

A graph analogue of the Grad-CAM is proposed to explain the results obtained from GCN-based models [36]. The first step is to compute the gradient of the class c with respect to the feature map F as:

(4) $$\alpha_{k}^{l,c}=\frac{1}{N}\sum_{n=1}^{N}\frac{\partial y^{c}}{\partial F_{k,n}^{l}},$$

where $F_{k,n}^{l}$ represents the k-th feature for node n at the l-th layer and $y^{c}$ represents the class score. The contribution vector (CAM) is calculated by a weighted combination of the forward activation maps at the l-th layer as:

(5) $$L_{Grad-CAM}^{c}\left(l\right)=ReLU\sum_{k}\alpha_{k}^{l,c}F_{k,n}^{l}$$

Therefore, the Grad-CAM method can be applied to a wide variety of CNN models, as well as various architectures for tasks, including image classification and image captioning. Moreover, Guided Grad-CAM, a combination of Grad-CAM and existing fine-grained visualizations, was proposed in the same paper to create a high-resolution and concept-specific visualization. It is capable of visualizing important regions of an image in high-resolution detail, which corresponds to any decision of interest. As a result, it makes up for the lack of showing fine-grained importance in Grad-CAM.

#### 4.1.3. Layer-Wise Relevance Propagation (LRP)

Bach et al. introduced layer-wise relevance propagation (LRP) to understand the classification decisions of the pixel-wise decomposition of nonlinear classifiers [37]. The LRP approach uses the output of the CNN, such as a classification score between 0 and 1, and iteratively backpropagates the output throughout the model structure. The realized backpropagation process follows the conservation property, i.e., the neurons received must be redistributed to lower layers in equal amounts, as shown below:

(6) $$\sum_{j}R_{j}=\sum_{k}R_{k.}$$

where *j* and *k* are neurons in different layers of the CNN. At the global level, it can be derived:

(7) $$\sum_{j}R_{j}=f(x),$$

where f(x) represents the output of the CNN model under the affection of *x*. It allows for the visualization of single pixels’ contributions to the predictions for CNNs. LRP also visualizes these pixel contributions as heatmaps. In each layer, the LRP approach assigns a relevance score to each of the input neurons from the previous layers, which equals the sum of the relevance score of its source neuron, in accordance with the conservation law.

#### 4.1.4. Trainable Attention

A trainable attention mechanism has been proposed to highlight which regions of the MRI images the CNN focuses on [38]. This trainable attention method displays where and to what extent the CNN ought to pay attention to the input images for the classification, and uses this attention highlight to further enhance the relevant regions and suppress the irrelevant regions.

#### 4.1.5. Guided Backpropagation

Springenberg et al. proposed a guided-backpropagation technique explanation, a gradient-based visualization technique that visualizes the gradient in relation to the images while backpropagating through the Relu activation function [39]. Guided backpropagation highlights the pixels that had the highest impact on the analysis output to create saliency maps. By adding guidance to the normal backpropagation, it limits the return of gradients less than 0, which corresponds to the undesirable parts of the original graph that weaken the features we want to visualize.

### 4.2. Model-Agnostic Explanation Methods

#### 4.2.1. Shapley Additive Explanations (SHAP)

Lundberg and Lee introduced the concept of SHapley Additive exPlanations (SHAP) to provide explanations for the predictions generated by machine learning models, using Shapley values from the game theory [40]. Shapley values reflect the marginal contributions of individual features to the model’s output separately to explicate why the model makes a certain prediction for a specific instance or sample [41]. By comparing the prediction with the average prediction distributed among the features, contrastive explanations can be derived. To approximate the Shapley values for CNNs, an innovative method named Deep SHAP has been developed. In MRI image analyses, Deep SHAP can be employed to identify which regions of the MRI image contribute positively or negatively to the output of the model.

#### 4.2.2. Local Interpretable Model-Agnostic Explanations (LIME)

Ribeiro et al. introduced Local Interpretable Model-agnostic Explanations (LIME), which interpret the predictions of DL models by approximating a CNN with a linear model [42]. The output of the complex model changes via perturbing the input data. The LIME method generates a new dataset (obtained by perturbing around the selected sample x) and then trains a simple model (interpretable model) on this new dataset, measuring the difference between the two models by the following objective function:

(8) $$\xi \left(x\right)=argmin\;L\left(f,g,\pi_{x}\right)+\sigma \left(g\right),$$

where *f* refers to the original model, *g* refers to the simple model, $\pi_{x}$ is the similarity of the perturbed input to the original input, and σ(g) is the complexity of model g. The $\pi_{x}(z)$ is used as a weight to guarantee that the explanations generated by the models with highly perturbed input data have less effect on the final explanation. The LIME algorithm uses the simpler linear model to learn the mapping between the perturbed input data, as well as the change in the output. Therefore, the above objective function can be optimized by means of a linear regression, as shown below:

(9) $$\xi \left(x\right)=\sum_{z,z^{\prime }\in Z}\pi_{x}\left(z\right){\left(f\left(z\right)-g\left(z^{\prime }\right)\right)}^{2}.$$

In MRI, the perturbations can be implemented by using super pixels to show which regions are of significance for explaining a classification output. There is an important premise that the simple model that LIME uses for its approximation must have the ability to distinguish between the positive and negative samples in the vicinity.

#### 4.2.3. Occlusion Sensitivity

Occlusion sensitivity is an analysis technique for visualizing which parts of an image are most important for classification tasks [43]. For classification methods, a natural question is whether a model actually determines the location of an object in an image or just uses the surrounding contextual information. To solve this, this perturbation-based technique perturbs the input image to assess the importance of certain regions of the target image. The idea behind this is that if the classification label is wrongly generated after a certain known key part of the input data (e.g., image) is occluded, the occluded part of the data is actually correctly learned/recognized by the model.

#### 4.2.4. Prediction Difference Analysis

For the purpose of visualizing the response of CNNs to a certain input, Zintgraf et al. adapted a prediction difference analysis method [44]. For each pixel considered to be an unknown feature, the prediction difference analysis method assigns a relevance value by measuring how the prediction changes. They expanded it by adding conditional sampling, in which only the analyzed pixels hard to predict were analyzed, simply by investigating the neighboring pixels. They also analyzed patches of connected pixels by adding a multivariable analysis.

## 5. XAI Applications in MRI

In MRI data analysis, XAI techniques have been used to provide explanations for DL methods performing classification and segmentation tasks. To conduct a study search, we searched for original published articles in the database of SpringerLink with the following criteria: (a) they contained the keywords “MRI”, “deep learning”, and “XAI”/“explainability”, AND (b) were written in English, AND (c) had been published since 2017. Then, we manually assessed the papers in the search results based on their relevance to the application of XAI in MRI image analysis. Finally, we included 56 of them in our survey (Table 1). We elaborate on the XAI applications in different parts of the human body in this section.

### 5.1. Brain

MRI provides a relatively high spatial resolution and non-invasive observation of neural activity, including changes in the brain’s oxygen levels, volume, connectivity, and cortical thickness. It contributes to the wide utilization of DL methods, as well as applications of XAI in neuroimaging.

#### 5.1.1. Brain Anatomical MRI

There are also multiple XAI methods utilized in MRI image analysis [34,44,45,46,47,48,49,50,51,52,53,54,55,56,57,58,59,60,61,62,63,64,65,66]. The value of T1-weighted MRI markers as adjuncts is being widely acknowledged by clinical assessment in the diagnosis and monitoring of progression. For example, T1-weighted MRI images become a significant part of the diagnoses and predictions of Alzheimer’s disease (AD). A wide range of model-specific XAI methods have gained highlighted performances in T1-weighted MRI image analysis. Shinde et al. built a novel CNN-based discriminative localization model named “high-resolution CAM”, based on the traditional CAM method [34]. They applied it to classify the ependymomas from a grade IV glioblastoma on T1-weighted contrast-enhanced (T1-CE) MRI data and to predict Parkinson’s disease from neuromelanin-sensitive MRI images. The method achieved a high accuracy for the diagnosis of mild cognitive impairments (MCI) and for yield-focused attention maps on the specific pathological locations related to MCI progression, which allows for more insights and a better understanding of the progression of MCI to AD. In another study, to assist clinicians in explaining the neural network decisions for diagnosing AD, Böhle et al. innovatively adapted the LRP technique to visualize the CNN decisions for AD based on T1-weighted MRI data [51]. LRP heatmaps can be interpreted as providing individual AD relevance as opposed to a general susceptibility for small variations in the input data. Shad et al. used the LIME method on a variety of CNN models, such as VGG, ResNet, and GoogLeNet, to look at T1-weighted MRI images [53].

Ahmad et al. developed a ResNet-based model that is capable of performing the accurate classification of brain tumors and tumor segmentation [54]. They relied on the CAM method to provide an explanation of their model. CAM heatmaps provide clinically meaningful insights into tumor regions, making the proposed model highly relevant in a clinical setting. Dubost et al. applied an attention maps approach to their 3D regression models, aiming at quantifying enlarged perivascular spaces (PVS) and the structural brain changes visible in MRIs and common in aging [58]. The attention maps were computed via guided backpropagation in terms of a visual and manual scoring of the PVS. This was the first qualitative evaluation to check whether a trained neural networks model was able to identify the structures of interest for PVS.

Furthermore, using both T1-weighted and T2-weighted MRI images, several studies have adapted the Grad-CAM algorithm to explain CNN models for the analysis of brain tumors [67,68,69,70,71,72]. For example, Windisch et al. implemented the Grad-CAM algorithm on ResNet to visualize the areas that their models used for outputting predictions in a brain tumor detection task [70]. Using state-of-the-art visualization attention maps, Zeineldin et al. established a new XAI framework named NeuroXAI based on the Grad-CAM algorithm for interpreting the behavior of CNNs and demonstrated the significance of incorporating XAI methods in brain tumor classification and segmentation tasks [71]. Intriguingly, they visualized a series of heatmaps generated by the Grad-CAM method in multiple individual layers of the U-Net model, demystifying the data flow within their segmentation model from the input MRI images to the output segmented masks.

#### 5.1.2. Brain Magnetic Resonance Angiography (MRA)

DL approaches have facilitated the diagnosis of vascular diseases and the prediction of brain ages using MRA [73,74,75]. Yin et al. made predictions of hemorrhagic and ischemia moyamoya disease (MMD) from brain TOF-based MRA images using ResNet pretrained on ImageNet [74]. They used the Grad-CAM technique to detect the ROIs, distinguishing the different MMD types. Nam et al. performed an age prediction from MRA images using a 3D CNN architecture [73]. They generated heatmaps of the MRA images with Grad-CAM and detected the important vascular structures related to aging. Mouches et al. predicted biological brain ages from a T1-weighted MRI and TOF-based MRA images with a multi-modal 3D CNN framework [75]. They drew saliency maps for both image modalities using SmoothGrad [76], a gradient-based interpretation model.

#### 5.1.3. Brain Diffusion Tensor Imaging (DTI)

Interpretable DL models have provided efficient diagnostic and prognostic models in the DTI field [77,78,79]. For example, Vidyadharan et al. calculated the four types of diffusion-based structural connectomes from a predefined atlas [78]. The structural connectomes were input into a deep CNN model to classify brain tumor grades. They then used Grad-CAM to reveal the pattern differences between low-grade glioma and high-grade glioma patients and found distinct patterns in the frontal, temporal, and parietal lobes. Velazquez et al., applied an ensemble model of a random forest and a CNN to classify early MCI and AD, using both DTI data and clinical features as inputs [79]. They also adopted Grad-CAM as the explanation of the white matter fiber differences between early MCI and AD. Huang et al. made graph classifications with their proposed GNN (MNC-Net) framework for early Parkinson’s disease (PD) diagnoses [77]. The model took the FA-based structural connectivity as node features and the sparse adjacency matrix as a graph. They identified the class-specific hub brain ROIs with the CAM technique. Occlusion sensitivity was also utilized to validate the detected ROIs.

#### 5.1.4. Brain Functional MRI (fMRI)

Recently, XAI techniques have been heavily involved in fMRI-related studies on revealing the dysfunctional ROIs related to brain diseases [80,81,82,83,84,85,86]. For example, Zhang et al. classified seven types of brain tasks using a knowledge-informed self-attention graph-pooling-based (SAGPool) GCN [81]. The model took the fMRI BOLD signals as node features and the binarized connectivity matrix of the functional connectomes as the graph for performing a graph classification. They explained the proposed method with the CAM method to select the important brain regions. Wen et al. used a prior brain structural learning-guided multi-view GCN framework to study autism spectrum disorder (ASD), where they formulated brain graph learning and multi-view learning to obtain the node features and the graph for each view and performed a graph classification [80]. They explained the model with CAM and identified the subnetworks and inter-subnetwork relationships related to ASD. Qu et al. used a multi-modal GCN model to predict cognitive scores with two-task fMRI paradigms, where the node features were the vectorized brain functional connectivity and the graph was the sparse binarized functional connectome [83]. The results were interpreted with gradient-weighted regression activation mapping (Grad-RAM, a variant of Grad-CAM) to detect the important brain regions.

### 5.2. Breast

Various studies have demonstrated that quantitative imaging such as dynamic contrast-enhanced MRI (DCE-MRI) could be used to characterize the various features related to tissue types (normal or abnormal tissue) [87]. DCE-MRI images of the breast were used to study the types of contrast enhancement kinetic curves, which are predictive of malignancy. DL models were available for providing better predictions for breast tumors based on the information provided by DCE-MRI. XAI methods are adapted to DL models to visualize the feature heatmaps for breast disease diagnoses [88,89,90,91]. For example, given T1-weighted MRI images being used as inputs, Adoui et al. developed a CNN model to predict responses to neoadjuvant chemotherapy, which aims to minimize the tumor size before surgery [89]. They visualized the most useful features contributing to classifying the pathological complete response (pCR) and non-pCR patients for the breast tumor prediction using the Grad-CAM method. In another study, for the purpose of gaining insight into the features learned by a CNN trained to classify estrogen receptor statuses (ER+ vs. ER−), based on DCE-MRI images of the breast, Papanastasopoulos et al. applied a model agnostic method called Integrated Gradients [92] to the ROIs from the training set [91]. Using attribution maps generated by this Integrated Gradients method, they identified the artifacts that may have interfered with the learning, which might provide guidance for improving our preprocessing steps and fine-tuning the DL models to learn the relevant features from DCE-MRI breast ROIs. Furthermore, they gained better insight into the imaging characteristics that may distinguish between ER+ and ER− patient cases.

### 5.3. Liver

The assessment of liver diseases is commonly involved in medical images. MRI plays an important role in liver disease detection and progression by assessing the liver’s morphology, signal intensity, and appearances following intravenous contrast material administration [93,94]. MRI images have been integrated with AI models to diagnose liver fibrosis and nonalcoholic fatty liver disease [95,96]. For example, Luetkens et al. developed DL models based on popular CNN architectures (ResNet50 and DesneNet121) to differentiate the etiology of liver cirrhosis using T2-weighted sMRI images. Then, they applied the Grad-CAM technique to explain the DL models’ decision process for classifying the liver cirrhosis as alcohol-related or non-alcohol related [97]. In another study, Li et al. developed DeepLiverNet, a multi-channel deep transfer learning convolutional neural network, to classify the severity of liver stiffness using axial anatomic T2-weighted abdominal MRI images and clinical features. They visualized the discriminative regions on T2-weighted liver images using the Grad-CAM technique to demystify the decision making process of the DeepLiverNet [98].

### 5.4. Musculoskeletal

MRI is effective in examining physical injuries or structural abnormalities. In lumbar MRI analysis, XAI methods have been used to provide a high level visualization of CNNs by generating saliency maps. For instance, Jamaludin et al. compared three backpropagation methods to extract the saliency maps that highlighted the pixels of T2-weighted sagittal spinal MRI images that had the highest impact on the localization of the spine pathologies [99]. They implemented contrastive excitation backpropagation and back-propagated up until the first convolution layer to achieve the best visual results.

MRI images are also a commonly used diagnostic examination for detecting severe or chronic internal injuries of the knee [100,101]. Using T1-weighted knee MRI images, Bien et al. developed an MRNet model to assist in the detection of general abnormalities and specific diagnoses such as meniscal tears. They generated heatmaps via the CAM technique to examine whether their model was capable of learning pertinent features from knee images [100].

### 5.5. Gastrointestinal

With advances in MRI, the difficulty of detecting gastrointestinal diseases declines. The diagnosis of gastrointestinal diseases may be further improved with AI and XAI methods. For example, Wang et al. developed a multi-branch cross-attention model to exploit the information contained in small T2-weighted MRI data sets of rectal cancer to learn discriminative features [102]. With the Grad-CAM technique, they confirmed that the highlighted ROIs in the MRI images were most helpful for predicting the Kirsten Rat Sarcoma virus mutation status, which is critical for clinicians to specify the treatment options for patients with rectal cancer.

### 5.6. Prostate

Prostate MRI is a desirable technique for an assessment of the extent of prostate cancer [103,104]. It is valuable to experts in making decisions on whether cancer has spread. In studying prostate MRI data, Hassan et al. used the LIME method to explain their VGG-based classification model for prostate cancer detection [104]. The LIME method explained the classification outcome via generating simulated images from the simplification of the original model, which initially located the regions that could be worthy of investigation given the input image. The automated computational approach of LIME identified the correct regions of interest that contained a malignant lesion with a uniform intact capsule, which explained why the model classified the patient as malignant. Moreover, the LIME algorithm could put emphasis on the ROIs that show a hyperechoic prostate with vertebral involvement, and the prostate’s ROIs annotated by radiologists could also be identified as important by the XAI approach.

### 5.7. Whole-Body

A whole-body MRI image looks at the body from head to toe. It is usually applied to find cancers across multiple tissues/organs. A whole-body MRI can also be applied to evaluate growth. Focused on images obtained via a whole-body MRI scan, Langner et al. trained a VGG-based CNN model on a large dataset for age prediction. They used the Grad-CAM method to generate saliency maps [105]. They not only examined the recurring patterns in a large number of individual saliency maps but also formed a combined visualization by aggregating the saliency maps to remove most of the noise, as well as patient-specific features, allowing for a comprehensive visualization of the most age-relevant anatomical structures.

### 5.8. Quantitative Analysis of Reviewed Applications

**Figure 3:**
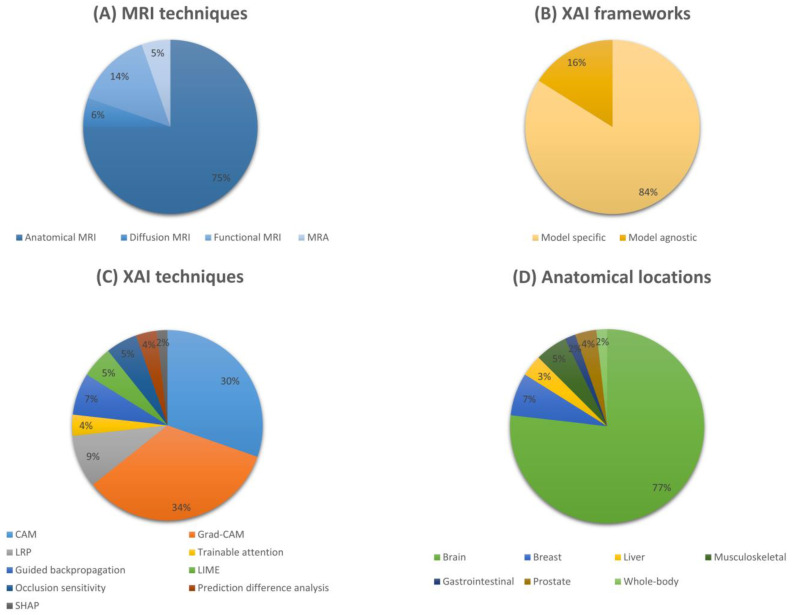
Quantitative analysis of non-exhaustive MRI studies using AI and XAI techniques since 2017. (**A**) Distribution of MRI techniques, (**B**) distribution of XAI frameworks, (**C**) distribution of XAI techniques, and (**D**) distribution of anatomical locations. MRA refers to Magnetic Resonance Angiography. CAM refers to Class Activation Mapping. Grad-CAM refers to Gradient-weighted Class Activation Mapping. LRP refers to Layer-wise Relevance Propagation. LIME refers to Local Interpretable Model-agnostic Explanations. SHAP refers to SHapley Additive exPlanations.

We conducted a quantitative analysis to investigate the reviewed MRI studies using AI and XAI techniques. We summarized the distributions of those pioneering works from four perspectives, including MRI techniques, XAI frameworks, XAI techniques, and anatomical locations. We display the analyzed distributions using pie charts in Figure 3. As shown in Figure 3A, 75% of the studies involved the utilization of anatomical MRI techniques, the most widely used MR imaging technique for various disease diagnoses and prognoses. Compared to this, other MRI modalities were only applied in relatively small portions of the research. Figure 3B demonstrates that 84% of the studies utilized XAI approaches that belong to the model-specific framework to demystify their models. Even though those model-agnostic approaches are considered “plug-and-play” tools, the trend shows that our research community prefers to employ model-specific approaches for their particular models. Figure 3C further illustrates a detailed XAI technique distribution. It is apparent that XAI in the MRI domain is dominated by CAM-based approaches. Grad-CAM was used in 34% of studies, while CAM was applied in 30% of them. Combined CAM-based methods occupied nearly two-thirds of the research. This is clearly due to the superior capability of Grad-CAM and CAM to explain CNN models. Finally, Figure 3D shows that most of these studies focused on brain MRI images. However, we believe this is likely because the MRI technique is the preferred imaging technique for investigating the human brain, which is irrelevant to XAI algorithms.

## 6. Evaluation of XAI in MRI

It is challenging and immature to evaluate if XAI algorithms are able to successfully explain DL models visually, due to the complex environmental and human factors. In recent years, multiple evaluation techniques for XAI methods have been proposed. Most of these evaluations are based on existing evaluation metrics, such as accuracy, stability, and plausibility.

### 6.1. Accuracy

Accuracy refers to how well XAI methods detect the relevant components of the input that the DL model trains on. In a recent study, Osman et al. evaluated XAI explanations on a synthetic dataset of rendered 3D shapes and generated an answering benchmark for relevant visual questions [106]. As for visual explanations, ground truth masks for evaluation need a 2D heatmap with a single channel, so they pooled the multiple channels of the original heatmaps, which mirror the shape of the model input down to a single-channel one. They also implemented two metrics, relevance mass accuracy and relevance rank accuracy, to evaluate several XAI methods. The former is calculated as the ratio of the sum of the relevance values within the ground truth mask over those in the whole image, and the latter measures how much high intensity relevance is within the ground truth. The mass and rank accuracy can be written as below:

(10) $$Mass\;Accuracy=\frac{R_{within}}{R_{total}},$$

(11) $$Rank\;Accuracy=\frac{\left|P_{top\;K}\cap GT\right|}{\left|GT\right|},$$

where $R_{within}=\sum_{\begin{array}{c} j=1\\ s.t.p_{j}\in GT \end{array}}^{J}R_{p_{j}},R_{total}=\sum_{i=1}^{N}R_{p_{i}}$, *GT* is the set of pixels lying within the ground truth mask, *J* is the number of pixels in the mask, $R_{p_{j}}$ is the relevance value of the pixel $p_{j}$, *N* is the total number of pixels in the image, *K* is the size of the ground truth mask, and $P_{top\;K}=\{p_{1},p_{2},\ldots ,p_{K}|R_{p_{1}}>R_{p_{2}}>\ldots >R_{p_{K}}\}$ represents the set of K highest relevance values. In their experiments, LRP outperformed previous XAI methods such as Integrated Gradients on both metrics.

### 6.2. Stability

Stability examines how slight perturbations in the input affect the explanation provided by XAI techniques [107]. For example, Douglas and Farahani examined the stability of XAI performance for neuroimaging [108]. They added slight Rician noise to the anatomical MRI data and obtained relevance heatmaps without greatly changing the CNN’s prediction performance for both the original and attacked images. Then, they conducted a relevance structural similarity analysis (RSSA) by implementing the method above to compare the contrast c, luminance l, and structural similarity s of the relevance heatmaps r between the original images $X_{r}$ and their corrupted counterpart $\tilde{X_{r}}$. The RSSA was computed as below:

(12) $$RSSA\left(\tilde{X_{r}},X_{r}\right)=\left[l\left(\tilde{X_{r}},X_{r}\right)\cdot c\left(\tilde{X_{r}},X_{r}\right)\cdot s\left(\tilde{X_{r}},X_{r}\right)\right].$$

They found that decomposition-based algorithms such as LRP were more stable than LIME.

### 6.3. Plausibility

Plausibility assesses how accordable the explanations generated by XAI algorithms are with their prior knowledge of the application [109]. Human-annotated ground truth is necessary for an agreeable XAI evaluation. For instance, Taghanaki et al. proposed an intersection over the predicted area (IoP), a plausible metric, to compare the heatmaps in a pneumonia disease detection task, which were generated by their InfoMask algorithm and traditional XAI algorithm, such as Grad-CAM [110]. By reflecting on what percentage of the region highlighted by XAI algorithms was inside the ground truth bounding box, the IoP provided a straightforward comparison of these XAI techniques. They observed that Grad-CAM tended to highlight larger regions of the input outside of the ground truth bounding box, while their proposed InfoMask generated contiguous attention regions, which were in most accordance with the ground truth box.

## 7. Conclusions

We present a survey on the recent advances in XAI algorithms utilized in MRI image analysis. From a systematic perspective, we first provide an introduction of MRI images and key DL models. We illustrate the frameworks of XAI methods and explain advanced XAI techniques. Furthermore, we outline the MRI-based applications that use AI models and XAI approaches. Finally, we discuss the common metrics in XAI evaluations. Our analysis reveals the insights of the MRI research domain into the current state of XAI techniques in MRI analysis.

AI will inevitably change MRI research. However, these ML and DL techniques are still subject to comprehensive interpretation/explanations for earning the trust of clinicians and human experts. To achieve further clinical translation, we need the assistance of more medical practitioners to evaluate these AI models. The XAI techniques reviewed in this work will be valuable tools for clinical MRI physicists, radiologists, and MRI technicians to work closely with. This will allow medical practitioners to have a better understanding of AI and its potential applications and limitations in clinical practice.

**Table 1:** Summary of recent XAI applications in various tissues/organs.

Location	Author	Year	Input	DL Method	Main XAI Method
Brain	Baumgartner et al. [45]	2018	T1 MRI	GAN	CAM
	Gao et al. [46]	2019	T1 MRI	DenseNet	CAM
	Li et al. [47]	2019	T1 MRI	CNN	CAM
	Shinde et al. [34]	2019	T1 MRI	CNN	CAM
	Shinde et al. [48]	2019	T1 MRI	ResNet	CAM
	Chakraborty et al. [49]	2020	T1 MRI	3D CNN	CAM
	Eitel et al. [50]	2019	T1 MRI	3D CNN	LRP
	Böhle et al. [51]	2019	T1 MRI	CNN	LRP
	Lian et al. [52]	2019	T1 MRI	FCN	Trainable attention
	Shad et al. [53]	2021	T1 MRI	VGG, ResNet, Inception	LIME
	Ahmad et al. [54]	2019	T2 MRI	Resnet	CAM
	Pominova et al. [55]	2018	T2 MRI	RCNN, etc.	Grad-CAM
	Liao et al. [56]	2020	T2 MRI	VGG16	Grad-CAM
	Grigorescu et al. [57]	2019	T2 MRI	3D CNN	LRP
	Dubost et al. [58]	2019	T2 MRI	ResNet	Guided backpropagation
	Dubost et al. [59]	2019	T2 MRI	3D Regression NN	Occlusion sensitivity
	Ceschin et al. [67]	2018	T1 + T2 MRI	3D CNN	CAM
	Pereira et al. [68]	2018	T1 + T2 MRI	CNN	Grad-CAM
	Natekar et al. [69]	2020	T1 + T2 MRI	Unet	Grad-CAM
	Windisch et al. [70]	2020	T1 + T2 MRI	Resnet	Grad-CAM
	Zeineldin et al. [71]	2022	T1 + T2 MRI	CNN	Grad-CAM
	Wei et al. [72]	2019	T1 + T2 MRI	GAN	Guided backpropagation
	Ng et al. [60]	2018	sMRI	CNN	CAM
	Yang et al. [61]	2022	sMRI	Unet	CAM, LIME, etc.
	Hilbert et al. [62]	2019	sMRI	Resnet	Grad-CAM
	Jain et al. [63]	2021	sMRI	GAN	Grad-CAM
	Dubost et al. [64]	2020	sMRI	Unet	Trainable attention
	Shahamat et al. [65]	2020	sMRI + fMRI	3D CNN	Occlusion sensitivity
	Zintgraf et al. [44]	2017	sMRI	CNN	Prediction difference analysis
	Seo et al. [66]	2020	sMRI	3D CNN	Prediction difference analysis
	Nam et al. [73]	2020	MRA	3D CNN	Grad-CAM
	Yin et al. [74]	2022	MRA	ResNet	Grad-CAM
	Mouches et al. [75]	2022	MRA	3D CNN	SmoothGrad
	Huang et al. [77]	2023	DTI	GNN	CAM, Occlusion sensitivity
	Vidyadharan et al. [78]	2022	DTI	CNN	Grad-CAM
	Velazquez et al. [79]	2022	DTI	CNN	Grad-CAM
	Wen et al. [80]	2022	fMRI	GCN	CAM
	Zhang et al. [81]	2023	fMRI	GCN	CAM
	Kim et al. [82]	2020	fMRI	GNN	Grad-CAM
	Qu et al. [83]	2021	fMRI	GCN	Grad-CAM
	Dang et al. [84]	2019	fMRI	MLP	LRP
	Xu et al. [85]	2019	fMRI	CNN	LRP
	Wang et al. [86]	2020	fMRI	CNN(DNN)	Guided backpropagation
Breast	Luo et al. [88]	2019	T1 MRI	3D ResNet	CAM
	Adoui et al. [89]	2020	T1 MRI	CNN	Grad-CAM
	Velden et al. [90]	2020	T1 MRI	3DregressionNN	SHAP
	Papanastasopoulos et al. [91]	2020	T1 MRI	DCNN	Integral Gradient
Liver	Li et al. [98]	2021	T2 MRI	DeepLiverNet	Grad-CAM
	Luetkens et al.	2022	T2 MRI	ResNet50, DesneNet121	Grad-CAM
Musculoskeletal	Bien et al. [100]	2018	T1 MRI + T2 MRI	MRNet	CAM
	Chang et al. [101]	2020	sMRI	CSN	CAM
	Jamaludin et al. [99]	2017	T2 MRI	VGG-M	Guided backpropagation
Gastrointestinal	Wang et al. [102]	2020	T2 MRI	CrossAttention	Grad-CAM
Prostate	Yang et al. [103]	2017	T2 MRI	multimodal CNN	CAM
	Hassan et al. [104]	2022	sMRI	VGG16	LIME
Whole-Body	Langner et al. [105]	2019	sMRI	CNN	Grad-CAM

## Data Availability

Not applicable.
